# Immunodiagnosis of Fascioliasis in Ruminants by ELISA Method: A Mini-Review

**DOI:** 10.21315/mjms2023.30.4.3

**Published:** 2023-08-24

**Authors:** Sudirman Nur Hafizah, Noor Jamil Noor Izani, Mohamad Ahmad Najib, Wan Abdul Wahab Wan-Nor-Amilah

**Affiliations:** 1 School of Health Sciences, Universiti Sains Malaysia, Kelantan, Malaysia; 2 Institute for Research in Molecular Medicine (INFORMM), Universiti Sains Malaysia, Kelantan, Malaysia

**Keywords:** ELISA, fascioliasis, Fasciola spp., immunodiagnosis, ruminants

## Abstract

Fascioliasis is an important zoonotic disease prevalent in domestic animals and it leads to socioeconomic impact in rural farming communities of the developing world. The gold standard diagnosis of ruminant fascioliasis involves coprological detection of *Fasciola* spp. eggs or recovery of flukes in infected livers. Coprological analysis is unreliable in the patent period of chronic infection, and even then, its sensitivity is relatively low. Robust diagnostic tools that can promptly and accurately detect an active infection are crucial to avoid complications and further losses in ruminant livestock productivity, as well as to preserve the livelihood of communities at risk. Immunodiagnosis determined by antibody and antigen detection in the sera and faeces of infected ruminants provides a valuable alternative to the parasitological diagnostic approach. This review discusses current developments in immunological techniques by enzyme-linked immunosorbent assay (ELISA) in the detection of ruminant fascioliasis and summarises the performance of various ELISAs in studies conducted to date. Indirect ELISAs demonstrated effective immunodiagnostic performance with high sensitivities and specificities. Cathepsin L ELISA is the most favourable antigen in serodiagnosis, among other recombinant and native proteins evaluated. Sandwich ELISA provides excellent sensitivity and specificity, which correlates well with the fluke burden. Utilising monoclonal antibodies in sandwich ELISA reduces the detection time and performance variations that commonly occur in polyclonal antibody ELISA.

## Introduction

Fascioliasis is a global helminthiasis caused by the trematode *Fasciola* spp., mainly *Fasciola hepatica* and *Fasciola gigantica*, which affect both animals and humans. In animals, it is a common infestation among ruminant livestock, such as cattle, sheep, goats and buffaloes (1–3). Its socioeconomic importance in communities whose livelihoods are highly dependent on livestock production is reflected by the substantial losses caused by reduced milk production in dairy animals, weight loss, disturbed growth rate, anaemia, breeding insufficiency, liver condemnation in abattoirs and mortality in acute cases of the infection (4–6), all of which amount to USD3.2 billion per year in cost (7).

Bovine fascioliasis has also gained interest with regard to its association with possible underestimation of bovine tuberculosis resulting from the downregulation of the T-helper type 1 (Th1) immune response that affects its diagnosis, particularly among the African cattle population (8). The vast distribution of fascioliasis includes many countries in different regions, such as Cameroon and Ethiopia in Africa; Peru and Bolivia in South America; Pakistan, Iran, Vietnam, Malaysia and Thailand in Asia; Ireland and Sweden in Europe; and Australia (7–11). As a plant-borne zoonotic disease, fascioliasis is typically transmitted via ingestion of aquatic vegetation contaminated with metacercariae (i.e. trematode cysts) released from intermediate lymnaeid vectors in freshwater. The parasites become excysted in the small intestine of the mammal host and penetrate the intestinal wall into the liver capsule, migrating through the liver parenchyma to develop into adult flukes in the biliary ducts (2).

Classical detection of fascioliasis requires either microscopic identification of *Fasciola* eggs in faeces by using sedimentation or flotation techniques, or recovery of the flukes from liver necropsy (8, 12, 13). Faecal detection of the parasite eggs is only possible in chronic infection (after 3 to 4 months) or within the patent period, whereas liver necropsy may overlook the early stage of *Fasciola* spp., thus leading to misdiagnosis (14). Both methods are time-consuming, labour-intensive and heavily rely on the skills of personnel involved in carrying out the procedures, which hinder their applicability in mass screening and surveillance settings (8, 12). Although liver necropsy has been shown to have near perfect sensitivity and specificity, faecal analysis has been known to exhibit varying sensitivities, to the extent of displaying false-negative findings in the presence of chronic or lighter infection (6, 8, 13, 15).

Immunodiagnostic techniques based on the detection of specific antibodies and antigens against *Fasciola* spp. in infected samples have been developed throughout the years as an alternative to coproscopy and liver necropsy techniques. The most important advantage of immunological approaches lies in their ability to detect infection in the prepatent period. Further, immunodiagnosis has been proposed as a promising approach for use in field studies among domestic animals or livestock due to its simpler procedures, proven diagnostic efficiency, and ability to yield fast results. This enables a more efficient and faster screening of large numbers of animals (16).

Enzyme-linked immunosorbent assay (ELISA) is the most widely used test in the serodiagnosis of veterinary fascioliasis, whereas several older techniques, such as immunoelectrophoresis, complement fixation, double diffusion and haemagglutination, are now rarely used due to their relative complexity (17). This mini-review focuses on current developments in different types of ELISA in detecting antibodies and antigens against *Fasciola* spp. and summarises the performance of various ELISAs in studies conducted to date.

### Enzyme-Linked Immunosorbent Assay

ELISA is the most famous and frequently used immunological-based assay for screening fascioliasis in ruminants. The most common configurations of these assays include indirect ELISA and sandwich ELISA. Indirect ELISA is used for the detection of antibodies against a specific antigen, in which the antigen is immobilised in a solid matrix to capture target antibodies. The sandwich ELISA, by contrast, utilises an immobilised anti-*Fasciola* antibody and once a specific antigen is bound to the antibody, an antigen–antibody complex is formed that triggers enzymatic cleavage of a chromogenic substrate, allowing detection of colour changes. Table 1 summarises the performances of indirect and sandwich ELISAs used in immunodiagnosis studies of ruminant fascioliasis.

### Indirect Enzyme-Linked Immunosorbent Assay

Infection by *Fasciola* spp. invokes humoural immune response, which is mediated by antibodies in the body of an infected host; thus, detection of antibodies is considered a reliable and suitable approach to the antemortem diagnosis of fascioliasis (18). Antibodies against *Fasciola* spp. are detectable in sera as early as 2 weeks post-infection (12). The antibodies remain at high levels in the different hosts up to 20 weeks after infection, even after the eradication of flukes. Indirect ELISA is used for the quantitative estimation of antibodies in serum and other body fluids.

Numerous antigens, such as crude fluke extracts, excretory/secretory (ES) products, tegumental antigens and recombinant proteins, have been used in ELISA development for human and ruminant fascioliasis detection. Most developed ELISAs described in past studies are based on ES antigenic products (4, 19–21), although they are known to cross-react with *Dictyocaulus viviparus, Nematodirus helvetianus* and *Ostertagia ostertagi* (13). Currently, many of the commercially available ELISAs employ ES antigens or antigens purified from ES products in their kits, such as the DRG ELISA kit, the Bio-X ELISA kit and the IDEXX (previously known as Pourquier) ELISA kit (22). A recent study compared the performance of an in-house ES ELISA kit to coprological analysis and the commercial Bio-X *Fasciola hepatica* ELISA, which employs cathepsin L1 antigen (Bio-X Diagnostics, Belgium), showing 98% sensitivity and 96% specificity of the in-house kit, as well as near perfect agreement with the results of the commercial kit (4).

The IDEXX ELISA kit (IDEXX, USA), which employs a component of ES products known as the F2 antigen, was described as exhibiting superior diagnostic performance with both sensitivity and specificity of 100%. By comparison, in-house ES ELISAs exhibited comparable sensitivities, although a cross-reaction with *D. viviparus* was observed, resulting in a specificity of 99.3% (23). The sensitivity and specificity of the in-house ES ELISAs can be affected by seasonal variability, with winter months having the highest performance and summer and autumn months having the lowest (13). Nonetheless, in a different study, the sensitivity and specificity of ES ELISA were reported as 65.3% and 65.2%, respectively, a startling contrast to the aforementioned findings, which could be due to a variety of factors, including uncertain co-infection status and low infection burden (8).

Cathepsin L, a highly immunogenic cysteine protease derived from ES proteins, is secreted by the parasite in all stages of its development in the host. The protease is thought to aid in the migration of parasites in the host and contribute to the immunomodulation of the host body response (18). The use of a more purified component has been recommended to reduce the incidence of cross-reactivity by ELISA and to prevent a false diagnosis (21). Dipstick ELISA that applies 28 kDa cathepsin L (FgCL3) was shown to excel in the prepatent serodiagnosis of naturally infected goats with 100% sensitivity and specificity (16). Similarly, recombinant cathepsin L ELISA in other ruminants demonstrated excellent performance, with sensitivity and specificity of at least 99% and 96.5%, respectively (24). In a more recent study, recombinant cathepsin L was found to be the most favourable antigen used in the ELISA serodiagnosis when compared to the other recombinant and native proteins tested, with 100% sensitivity and 97% specificity (25).

Tegumental antigens (i.e. components that originate from tegument of *Fasciola* spp.) are another group of proteins that have been evaluated in ELISA and were found to be successful in detecting antibodies, despite having cross-reactivity (26). A previous study isolated a purified 16.5 kDa tegumental antigen (FhTP16.5) that reacted with the sera of infected rabbits and humans, although cross-reactions with other infections could not be assessed due to a lack of available samples (27).

Immunoassays based on antibody detection have been frequently employed in seroprevalence studies, some of which also include baseline analysis for future assessments of risk factors (11, 28, 29). Such an assay was tested in the evaluation of flukicide efficacy and was found to be impractical for this purpose, as the post-treatment antibody levels did not decrease (12, 30, 31). ELISA based on antibody detection is also limited by its inability to distinguish an active infection from a resolving one or past exposure to the parasite, thus requiring careful interpretation of the results. Despite these limitations, indirect ELISAs have demonstrated effective immunodiagnostic performance, with high sensitivities and specificities in previous studies (19, 13, 32).

### Sandwich Enzyme-Linked Immunosorbent Assay

Detection of *Fasciola* spp. antigens by sandwich ELISA is favoured due to its capacity to accurately reflect the presence of ongoing infection, as opposed to indirect ELISA. Sandwich ELISAs are frequently based on monoclonal antibodies (mAbs) to minimise errors caused by batch-to-batch variations common in polyclonal antibody production. The use of mAbs in this assay improves the sensitivity and specificity of immunodiagnosis (21, 33). A commercialised capture sandwich ELISA, Bio K 201 kit (Bio X Diagnostics, Belgium) employing MM3 mAb for the detection of *Fasciola* spp. ES coproantigens has been developed following a study that established its excellent diagnostic performance and positive correlation with fluke burden (34). A more recent study confirmed these initial findings, reporting an absence of cross-reactivity with *Paramphistomum cervi* and *Taenia hydatigena* (35).

Subsequent studies using the same commercial kit, however, have yielded varying degrees of sensitivity and specificity in ruminant infections. A longitudinal study conducted in Scotland reported a significantly lower sensitivity of 77% and specificity of 99% (13). The sensitivity was improved with a modified cut-off point but only up to 80% compared to the value recommended by the manufacturer. In another study from Western Australia, similar results were obtained in the evaluation of the ELISA, which could only be improved with modified cut-off points, from 88% to 100%, 80% to 87% and 9% to 27% in sheep, cattle and horse infections, respectively. However, the degree of specificity was maintained in all scenarios at 99% (36).

*Fasciola* spp. coproantigens are detectable by ELISA at 6 weeks post-infection in most ruminants, which is later than antibody detection (6, 15). This highlights the assay’s lack of reliability in detecting the presence of immature flukes, although it performs adequately in adult infections (37). False-negatives have also been noted in light infections, as the dilution of coproantigens in the sample compromises the absorbance reading, resulting in poor detection. Coproantigen levels have been shown to correlate strongly with the fluke burden in animal hosts (15). A later study, however, reported a contradictory conclusion, which could be attributed to the low burden of flukes on the animals used in the study (38).

ELISA based on the detection of circulating antigens has also been shown to be a reliable approach and allows earlier identification of infection compared to coproantigen ELISA (39, 40). Sandwich ELISA based on specific mAb against *F. gigantica* circulating surface tegument antigen detected the antigen as early as 2 to 3 weeks post-infection. Although its sensitivity was determined to be 100%, cross-reactions were observed with other trematode species (33). An in-house ELISA that used mAb against cathepsin B3 protease in the sera of naturally infected cattle yielded 96.7% sensitivity and 100% specificity (39). A subsequent study on the detection of cathepsin L reported exceptional results, with 98.3% sensitivity and 100% specificity, as well as an improved detection limit (40). In both studies, the circulating antigens were detectable on day 1 post-infection. Coproantigen detection by ELISA is generally applied in efficacy assessments of flukicides, such as triclabendazole and albendazole, in domestic ruminants. The advantages and limitations of both indirect and sandwich ELISAs are summarised in Table 2.

## Conclusion

Fascioliasis is a significant concern in the livestock industry due to its substantial impact on the control and treatment-associated costs incurred, as well as the loss in milk and meat production. Indirect and sandwich ELISA have clear advantages over conventional diagnosis of fascioliasis by parasitological approach in terms of superior diagnostic efficiency and earlier detection in the prepatent period. Although indirect ELISAs have been broadly employed in diagnosis and field screenings, there is still evidence of variable findings when performed by different researchers and in different regions and/or host ruminant species. MM3, F2, cathepsin L and cathepsin B3 have demonstrated improved sensitivity and specificity in diagnosis using ELISA; however, an optimal and well-characterised antigen that could produce consistent results has yet to be discovered.

Sandwich ELISA based on coproantigen detection using specific monoclonal antibodies has provided an option for establishing a potential standardised methodology for prevalence and treatment efficacy research, although it is highly recommended that cut-off points be modified based on the local ruminant population prior to screening. However, light infections by *Fasciola* spp. further complicate diagnosis in ruminants and result in false negatives. Therefore, to improve diagnostic sensitivity, future research should investigate optimal antigen and monoclonal antibody production for antibody and antigen detection immunoassays, respectively.

## Figures and Tables

**Table 1:** Summary of indirect and sandwich ELISA methods used in the immunodiagnosis of ruminant fascioliasis

Test performance of different antigens used in indirect ELISA	Test performance of different antibodies used in sandwich ELISA
Crude excretory/secretory antigens (ES Ag) Lower sensitivity and specificity Exhibit cross reactivity (4, 19–21) F2 antigen (a component of ES proteins) Sensitivity and specificity in the range of 65% to 100% Exhibits lesser cross-reactivity (8, 23) Cathepsin L1 (cysteine protease derived ES proteins) Recombinant CL ELISA demonstrated excellent sensitivity (99%) and specificity (96.5%) (24) Dipstick ELISA (28 kDa cathepsin L; FgCL3) demonstrated excellent prepatent serodiagnosis of naturally infected ruminant (16) Tegumental antigen (a purified 16.5 kDa tegumental antigen (FhTP16.5) Successful detection of antibodies Exhibits cross-reactivity (26, 27)	MM3 monoclonal antibody (mAb) for the detection of *Fasciola* spp. ES coproantigens (6, 15, 37) Exhibits a positive correlation with fluke burden (34) Absence of cross-reactivity (35) Variable sensitivity and specificity in ruminant infections (13) Specificity remains at 99% (36) The coproantigens are detectable by ELISA from 6 weeks post-infection (later than antibody detection) in most ruminants (6, 15) Specific mAb against *F. gigantica* circulating surface tegument antigen Allows earlier and reliable identification of infection compared to coproantigen ELISA (39, 40) Excellent sensitivity (100%) Exhibits cross-reactions with other trematode species (33) ELISA mAb against cathepsin B3 protease in sera Sensitivity (96.7%–98.3%) Specificity (100%) Improved detection time of circulating antigens in naturally infected cattle from day 1 of post-exposure (39, 40)

**Table 2:** Advantages and limitations of indirect and sandwich ELISA methods in the immunodiagnosis of ruminant fascioliasis

	Indirect ELISAs (detection of antibodies)	Sandwich ELISA (detection of antigens)
Advantages	Demonstrates effective immunodiagnostic performance with high sensitivities and specificities (19, 23, 32) Recombinant CL ELISA is the most favourable antigen in serodiagnosis among other recombinant and native proteins evaluated (25) 100% sensitivity and specificity in prepatent serodiagnosis (16)	Excellent sensitivity and specificity Correlates with fluke burden (34) Accurately reflects the presence of ongoing infection Monoclonal antibodies shorten detection time (from day 1 of post-infection) (40) Monoclonal antibodies reduce variations common in polyclonal antibody ELISA
Limitations	Inability to distinguish active infection from past exposure to the parasite	Coproantigens detection is longer (from 6 weeks post-infection) Varying degree of cross-reactivity is seen
